# Force regulated dynamics of RPA on a DNA fork

**DOI:** 10.1093/nar/gkw187

**Published:** 2016-03-25

**Authors:** Felix E. Kemmerich, Peter Daldrop, Cosimo Pinto, Maryna Levikova, Petr Cejka, Ralf Seidel

**Affiliations:** 1 Institute of Experimental Physics I, Universität Leipzig, Linnéstr. 5, 04103 Leipzig, Germany; 2 Institute for Molecular Cell Biology, University of Münster, Schlossplatz 5, D-48149 Münster, Germany; 3 Institute of Molecular Cancer Research, University of Zurich, Winterthurerstrasse 190, CH-8057 Zürich, Switzerland

## Abstract

Replication protein A (RPA) is a single-stranded DNA binding protein, involved in most aspects of eukaryotic DNA metabolism. Here, we study the behavior of RPA on a DNA substrate that mimics a replication fork. Using magnetic tweezers we show that both yeast and human RPA can open forked DNA when sufficient external tension is applied. In contrast, at low force, RPA becomes rapidly displaced by the rehybridization of the DNA fork. This process appears to be governed by the binding or the release of an RPA microdomain (toehold) of only few base-pairs length. This gives rise to an extremely rapid exchange dynamics of RPA at the fork. Fork rezipping rates reach up to hundreds of base-pairs per second, being orders of magnitude faster than RPA dissociation from ssDNA alone. Additionally, we show that RPA undergoes diffusive motion on ssDNA, such that it can be pushed over long distances by a rezipping fork. Generally the behavior of both human and yeast RPA homologs is very similar. However, in contrast to yeast RPA, the dissociation of human RPA from ssDNA is greatly reduced at low Mg^2+^ concentrations, such that human RPA can melt DNA in absence of force.

## INTRODUCTION

Replication protein A (RPA) is a highly ubiquitous (1), heterotrimeric (2), protein essential in virtually all aspects of eukaryotic DNA processing involving single-stranded DNA (ssDNA) intermediates (3). Due to the strong binding of RPA to ssDNA (2,4–6). RPA was originally thought to solely prevent the formation of secondary structures and confer protection from nucleolytic degradation. However, strong evidence for direct interactions with specific protein partners has been reported (7–10), and a new paradigm emerged. RPA is now thought to act additionally as a scaffold for the recruitment of other DNA processing enzymes on ssDNA intermediates, in order to channel the processing along specific pathways (11,12). RPA coated ssDNA for example signals the presence of DNA damage to the checkpoint machinery through direct binding of ATR-interacting protein (ATRIP) (8,13,14). From this viewpoint, the boundaries between ssDNA and double-stranded DNA (dsDNA), i.e. the interface upon which a multitude of DNA processing factors are acting, are of particular interest. Here, the binding and release of RPA must be highly dynamic, and organized in such a way that the DNA can be rapidly made accessible to subsequent processing machinery.

The importance of ssDNA–dsDNA boundaries is also highlighted by the fact that despite the low affinity toward dsDNA (15), RPA binds appreciably to ssDNA stretches exposed upon dsDNA damage (16,17), is able to disrupt partially dsDNA structures such as triplexes (18), tetraplexes (19,20) and suppresses formation of secondary structures such as hairpins (11). Under certain circumstances, the ATP-independent melting of dsDNA by RPA has also been shown (21–23), where it was proposed that the observed duplex destabilization proceeds by trapping fluctuations of the helix (23).

Several recent studies have advanced our understanding of the molecular mechanisms that may control the coordination of RPA by employing single-molecule analysis techniques: (i) Using single-molecule DNA supercoiling experiments in magnetic tweezers it was shown that RPA can bind to transiently forming bubbles in the DNA duplex in a torque-dependent manner (24). (ii) Single-molecule imaging of fluorescent RPA has shown that RPA bound ssDNA may undergo more rapid exchange in presence of free RPA in solution (25). (iii) Using a combination of single-molecule fluorescence techniques it was found that under high salt conditions RPA may diffuse/slide along ssDNA (26), suggesting the intriguing possibility that in this way access to the DNA is provided to other enzymes. Recently, Chen and Wold (12) pointed out that central to all of these single-molecule studies is the emerging view that RPA binding is highly dynamic and that microscopic rearrangements of the RPA DNA binding domains (DBDs) are underlying the observed dynamics. However, it was also emphasized that more work is required to fully understand the rich dynamics of RPA in complex with various DNA structures.

Here, we investigate in detail the dynamics of RPA at the boundary of ssDNA and dsDNA such as present at a replication fork. We utilize magnetic tweezers that allow precise manipulation and length determination of immobilized DNA substrates via an attached magnetic microsphere (27). At the single-molecule level they support the study of fast dynamic processes, and allow dissecting inherent molecular variation with spatial resolution on the scale of one base-pair (bp) (27).

We have characterized the force-dependent binding dynamics of RPA from human and budding yeast (*Saccharomyces cerevisiae*), henceforth referred to as hRPA and yRPA (see Materials and Methods for details) on a DNA fork down to single protein association events. The interplay between RPA, the forked DNA substrate and force tightly regulates the balance of RPA binding and the opening and closing of the fork. Our results indicate that RPA uses a ‘toehold’-like mechanism to trap small transient openings of the DNA helix with a microdomain, which are then expanded as the full protein wedges in to bind. Similarly, RPA displacement by the rezipping fork first occurs through an initial rate-limiting displacement of a toehold. This gives rise to a very rapid helix rezipping upon which RPA dissociates much faster than on ssDNA. Facilitated by our observation of both RPA homologs, we can confirm that the described behavior is a general trait of RPA across different organisms. Thus, while RPA protects ssDNA rather firmly and statically, it is extremely dynamic at DNA processing sites. This is additionally supported by the observation that a DNA fork can slide/push RPA upon rezipping.

## MATERIALS AND METHODS

### DNA substrates

A DNA substrate containing a 488 bp long hairpin was prepared as described previously (28). The 5′-end of the hairpin carried a single biotin modification, while the 3′-end was linked through a 60 nt ssDNA spacer to a 1 kb dsDNA spacer, followed by a 600 bp digoxigenin-modified attachment handle.

Preparation of the flapped DNA duplex substrate has been described previously (29,30). Central part is a 6.1 kb unmodified dsDNA with a flap located 1 kb from its proximal DNA end. Approximately 600 bp attachment handles carrying multiple digoxigenins and biotins were attached to the 6.1 kb fragment at its flap-proximal and distal ends, respectively.

### Recombinant proteins

yRPA and hRPA were recombinantly expressed and purified as described previously (31,32). In brief, yRPA was expressed in the yeast strain BJ5464 containing three plasmids, coding for subunits Rfa1, Rfa2 and Rfa3, respectively. Cells were lysed and yeast RPA was purified by affinity on a ssDNA cellulose column (USB corporation, Cleveland, USA) and by ion exchange chromatography using a HiTrap Q column (GE Healthcare, Little Chalfont, UK). Human RPA was expressed from the p11d-tRPA vector (32) in BL21 *E. coli* cells and purified by chromatography using HiTrap Blue and HiTrap Q columns (GE Healthcare, Little Chalfont, UK).

### Magnetic tweezers experiments

For the single molecule experiments a custom magnetic tweezers setup was utilized (27,33). Magnetic tweezers experiments were conducted at room temperature using flow cells assembled from two coverslips (Menzel, Braunschweig, Germany) that were separated by a layer of Parafilm (Bemis, Oshkosh, USA) into which a sample chamber was cut out. The bottom coverslip was coated with polystyrene (Sigma-Aldrich, St. Louis, USA). Three micrometers carboxyl-modified latex beads (Life Technologies, Darmstadt, Germany) that served as reference, were attached to the bottom slide of the mounted flow cell by incubation in 1 M NaCl for 1 h. Subsequently, anti-digoxigenin antibodies (Roche, Penzberg, Germany) were allowed to unspecifically bind to the coated surface of the flow cell, by incubation at a concentration of 50 μg/ml for 1 h at room temperature. Subsequently, the flow cell was passivated by overnight incubation with 10 mg/ml bovine serum albumin (BSA, New England Biolabs, Ipswich, USA). DNA constructs were bound to streptavidin-coated M280 magnetic beads (Life Technologies, Darmstadt, Germany) and then flushed into the flow cell. After allowing them to bind for ∼5 min, excess beads were washed out with phosphate buffered saline (PBS) solution rendering the sample chamber ready for experiments. The positions of reference and DNA-attached beads were tracked in all three dimensions at 300 Hz using videomicroscopy and real-time GPU-accelerated image analysis (27). Typically 15-20 beads were evaluated in parallel. Forces were calibrated using a recent methodology that supports the usage of short molecules and high forces (34). Experiments were conducted in 50 mM Tris acetate pH 7.5 supplemented with magnesium acetate in concentrations as described in the results. Data were analyzed in Labview (National Instruments, Austin, USA), Origin 9.1 (OriginLab, Northampton, USA) and Matlab (MathWorks, Natick, USA). Length changes measured in nm for opening of the hairpin or melting of the nicked DNA construct were converted into the number of opened bp (Supplementary Data).

### Gel-based DNA melting experiments

As substrate for dsDNA melting experiments by gel electrophoresis, double-stranded Lambda DNA was digested with HindIII (New England Biolabs, Ipswich, United States) and 3′-labeled with [α^32^P]dATP using the Large Klenow fragment of DNA polymerase I (New England Biolabs, Ipswich, USA). Unincorporated nucleotides were removed using MicroSpin G25 columns (GE Healthcare, Little Chalfont, UK). The resulting labelled DNA fragments had short 3 nt long 5′ ssDNA tails. Experiments were performed in a 15 μl volume in 25 mM Tris-acetate pH 7.5, 1 mM dithiotreitol, 0.1 mg/ml BSA, 1 mM phosphoenolpyruvate, 0.02 U/ml pyruvate kinase (Sigma-Aldrich, St. Louis, USA), 0.15 nM DNA substrate and magnesium acetate and recombinant proteins as indicated. RPA was present in the reactions at saturating concentrations corresponding to an excess over DNA as indicated, assuming all DNA was single-stranded and a DNA-binding site size of 25 nt for human RPA and of 20 nt for yeast RPA. A complete 100% DNA saturation thus corresponds to 576 nM hRPA or 720 nM yRPA. Reactions were incubated at 37°C or 30°C as indicated for 30 min, and then terminated by adding 5 μl stop buffer (150 mM EDTA, 2% SDS, 30% glycerol and 0.01% bromophenol blue), and analyzed on 1% agarose gels in 1X TAE buffer. Gels were dried, exposed to a storage phosphor screen and analyzed on a Typhoon phosphor imager (GE Healthcare, Little Chalfont, UK).

## RESULTS

### RPA association and dissociation at a DNA fork

**Figure 1.**
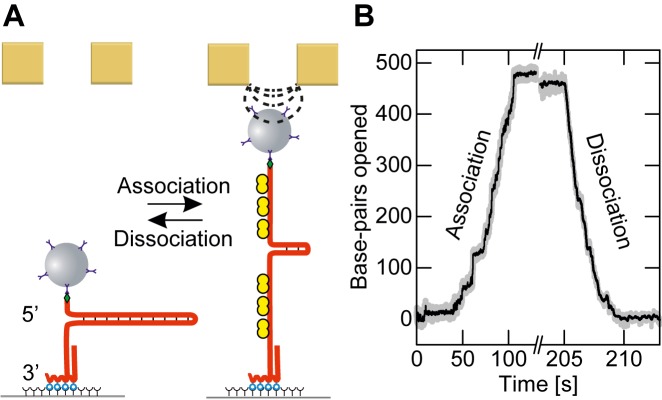
Force controlled association and dissociation of RPA on a DNA fork substrate. (**A**) Schematic of the experiment: a 488 bp long DNA hairpin substrate (red) is immobilized onto a glass surface and tethered to a magnetic bead. When sufficient force is applied to the bead, RPA (yellow) can bind to the ssDNA/dsDNA interface at the fork. As a result, the hairpin is opened to accommodate the entire RPA heterotrimer. Further association proceeds by the contiguous binding of RPA to the fork until the hairpin is fully opened. Upon lowering the magnetic force the DNA helix refolds reversibly and RPA dissociates from the ssDNA. (**B**) Example time trace of force controlled yRPA association and dissociation (in presence of 20 nM yRPA and 3 mM Mg^2+^). At a force of 13.2 pN, sequential binding of RPA opens the hairpin with a rate of 4.7 bp/s, until it is fully opened and completely covered with RPA. Lowering the force to 4.5 pN causes dissociation of RPA evident in rapid refolding of the hairpin with a rate of 107 bp/s.

We measured the association and dissociation of RPA on a 488 bp long DNA hairpin substrate using magnetic tweezers. One end of the hairpin was immobilized via a dsDNA spacer at the bottom surface of a fluidic cell. The other end was tethered to a 2.8 μm magnetic bead (Figure 1A). A set of permanent magnets was mounted on a movable stage above the fluidic-cell, such that the magnetic force acting on the bead could be controlled by lowering or raising the magnets (see Materials and Methods for details).

The DNA hairpin substrate can be opened mechanically by applying sufficient force to the bead. At forces above a critical force, which will be referred to as unzipping force, a series of sudden, well-defined transitions in DNA extension occurs, amounting to about 475 nm from the fully-closed to the fully-open state over the course of about 1 s (see Supplementary Figure S1). In contrast, after adding yRPA a gradual opening of the hairpin at forces well below the unzipping force was observed. In this case, the opening process lasted for much longer time-scales and continued until the hairpin was extended to the fully-opened state (Figure 1B). Upon reduction of the applied force, a gradual reversion to the closed state took place. We interpret these gradual transitions as the result of RPA binding to the fork of the hairpin. The sequential association of more RPA opened the hairpin and generated RPA-covered ssDNA. Upon lowering the force, RPA is displaced by the rezipping of the DNA (Figure 1A). The slopes of both association and dissociation were approximately constant throughout the complete hairpin opening or closing process, irrespective of length of dsDNA remaining. This suggests that RPA binding occurs only at the fork, and in a contiguous manner with respect to the previously bound RPA. We emphasize that the process is fully reversible and the same substrate molecule can be opened and closed multiple times without systematic alteration of the resulting curves.

### Force dependence of RPA binding at the fork

**Figure 2.**
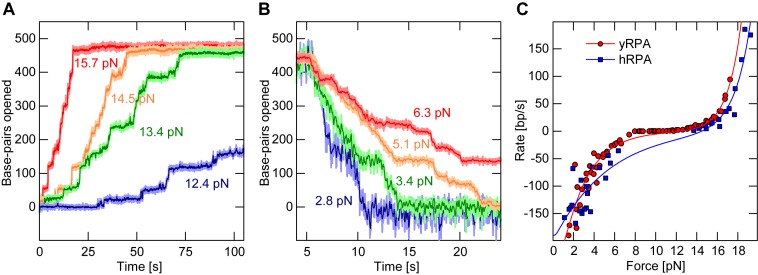
Force dependence of the RPA association/dissociation kinetics at the fork. (**A**) Example time traces of yRPA (20 nM in presence of 3 mM Mg^2+^) association on the DNA hairpin at different forces. The DNA hairpin sequentially opened due to successive association events of RPA. With increasing forces the overall association rate becomes faster, ranging from 2.8 bp/s at 12.4 pN (blue) to 36.6 bp/s at 15.7 pN (red). (**B**) yRPA (20 nM in presence of 3 mM Mg^2+^) dissociation time traces for varying force. Following a complete coverage of RPA on the DNA hairpin substrate, the force was lowered to the indicated values causing the hairpin to close and RPA to dissociate. The rate of dissociation is much faster than association and ranges from 107 bp/s at 2.8 pN (blue) to 16.2 bp/s at 6.3 pN (red). (**C**) Association and dissociation rates as a function of force, obtained by tracking multiple DNA tethered magnetic beads in parallel for yRPA (red circles, 20 nM in presence of 3 mM Mg^2+^) or by tracking a single bead with hRPA (blue squares, 50 nM in presence of 10 mM Mg^2+^). The data were fit using Equation 1 (red and blue lines for yRPA and hRPA, respectively) with fit parameters listed in Table 1.

Next we investigated the influence of the applied force on the association and dissociation rates of RPA on the DNA hairpin substrate. These rates showed a strong dependence on the applied force. Above 11 pN we observed a gradual opening of the hairpin and the rate of opening increased with stronger force (Figure 2A). When the force acting on such an RPA covered, open hairpin, was reduced below 11 pN, we observed a gradual closing of the hairpin. The observed closing rates were the faster the lower the applied force (Figure 2B).

Plotting the rates of hairpin opening and closing against the applied force, it became apparent that both the association and dissociation rates varied exponentially with force as shown in Figure 2C. We devised a model in which the net rate of RPA binding to the DNA is the difference between the rates of force-dependent RPA association and RPA dissociation from the fork. Each rate is expressed as an exponential Arrhenius term as obtained from transition-state theory in which the applied force *F* scales with height of the energetic barrier to the transition state:

(1) \begin{eqnarray*}v_{{\rm net}}&=&k_{{\rm on}}\,\exp \left( \frac{\left(c_F\cdot F-c_{{\rm unz}}\cdot F_{{\rm unz}}\right) \Delta z_{{\rm on}}}{k_BT} \right) \nonumber \\ &-&k_{\mathit {{\rm off}}}\,\exp \left(-\frac{c_F\cdot F\cdot \Delta z_{\mathit {{\rm off}}}}{k_BT} \right) \end{eqnarray*}

Pre-exponential factors *k*_on_ and $k_{\mathit {{\rm off}}}$ describe the expected rates for association and dissociation at the unzipping force *F*_unz_ or at zero force, respectively. The second pair of fit parameters (Δ$z$_on_ and $\Delta z_{\mathit {{\rm off}}}$) corresponds to the distance of the initial state (before association/dissociation of a new RPA) to the transition state along the relevant reaction coordinate, in this case the number of bp along the DNA hairpin. For association, Δ$z$_on_ thus corresponds to the number of bp that need to open spontaneously to accommodate a sufficiently long part of an RPA complex, such that the full complex can subsequently bind. For dissociation $\Delta z_{\mathit {{\rm off}}}$ is the number of bp that need to rezip and displace part of the RPA complex, to allow full complex dissociation. The factor *c* converts the number of bp into a DNA extension change, i.e. a length. This conversion is a function of force (see Supplementary Figure S2) and was determined as described in the Supplementary Information. The model describes the force-dependent rates well, as evident from the fits to the data (Figure 2C). At concentrations of 20 nM yRPA and 3 mM Mg^2+^ the fit yields a fast hairpin closure rate of at zero force of $k_{\mathit {{\rm off}}}=239\,\pm \,14$ bp/s. Furthermore, values of $\Delta z_{\mathit {{\rm on}}}=3.3\,\pm\,0.6$ bp and $\Delta z_{\mathit {{\rm off}}}=1.6\,\pm\,0.1$ bp were obtained, indicating that spontaneous helix opening or partial RPA displacement amounting to only a few bp is required to overcome the transition state for RPA association or dissociation, respectively. This is analogous to the toehold mechanism in DNA nanotechnology (35), where the association of a small protein microdomain (toehold) is the rate-limiting step for full protein binding and further helix opening. Conversely, the disengagement of a terminal microdomain is rate-limiting for helix rezipping.

The fit also provided the hairpin opening rate $k_{\mathit {{\rm on}}}=180\,\pm \,42$ bp/s at the unzipping force of *F*_unz_ = 18.2 pN. At the unzipping force the hairpin no longer imposes an energetic hurdle for RPA association, such that the extrapolated rate should approximate the RPA association to free ssDNA. Hairpin opening rates at a given force increased linearly with the RPA concentration (between 5 and 50 nM, see Supplementary Figure S5), allowing us to calculate standardized (per nM) rates (see Table 1 for the full set of fit parameters).

**Table 1.** Fit parameters for yRPA and hRPA association and dissociation kinetics

	yRPA			hRPA		
	*1 mM Mg^2+^*	*3 mM Mg^2+^*	*10 mM Mg^2+^*	*3 mM Mg^2+^*	*5 mM Mg^2+^*	*10 mM Mg^2+^*
$k_{\mathit {{\rm on}}}$						
[bp s^-1^ nM^-1^]	34.2 ± 16.9	9.0 ± 2.1	18.2 ± 8.9	2.1 ± 0.3	3.2 ± 0.4	6.2 ± 1.1
$k_{\mathit {{\rm off}}}$ [bp s^-1^]	233 ± 19	239 ± 14	336 ± 51	37 ± 7	109 ± 28	189 ± 23
$\Delta z_{\mathit {{\rm on}}}$[bp]	5.0 ± 1.0	3.3 ± 0.6	2.4 ± 0.7	1.6 ± 0.6	2.1 ± 0.4	2.5 ± 0.5
$\Delta z_{\mathit {{\rm off}}}$ [bp]	1.8 ± 0.2	1.6 ± 0.1	1.8 ± 0.2	0.6 ± 0.5	1.1 ± 0.5	0.9 ± 0.2
$F_{\mathit {{\rm unz}}}$ [pN]	17.8	18.2	19.9	18.2	18.8	19.9
$F_{\mathit {{\rm equi}}}$[pN]	12.4	12.4	11.1	11.8	12.2	14.5

$k_{\mathit {{\rm on}}}$ and $k_{\mathit {{\rm off}}}$ are the rates of association and dissociation at the unzipping force $F_{\mathit {{\rm unz}}}$ or zero force, respectively. $\Delta z_{\mathit {{\rm on}}}$ and $\Delta z_{\mathit {{\rm off}}}$ are the transition state distances for binding or dissociation of an RPA heterotrimer in bp. The parameters were obtained by fitting the force-dependent association and dissociation rates of yRPA and hRPA on the 488 bp DNA hairpin, to a model comprised of two Arrhenius terms (see Equation 1).

Taken together, the data so far show that in the absence of force, dissociation dominates such that the closed hairpin state is favored. Increasing the forces ultimately tips the scales and hairpin opening becomes favored. From our model parameters one can also calculate the force *F*_equi_ (see Table 1), at which hairpin opening and closing rates are at equilibrium, which is 12.4 pN for 20 nM yRPA in 3 mM Mg^2+^.

To test whether hairpin opening/closing driven by the association/dissociation of microdomains is a general property of RPA, we repeated our experiments with hRPA. Similarly to yRPA, a force-dependent opening and closing of the DNA hairpin due to the association and dissociation of hRPA at the fork was observed (Figure 2C). Our model (Equation 1) also described the hRPA data well, and a fit to the data yielded parameters that were comparable to yRPA (Table 1). Most importantly the transition state distances again amounted to only few bp also for hRPA, which suggests that both RPA homologs use a toehold mechanism for binding/dissociation at a DNA junction.

### RPA binding on a DNA duplex substrate

**Figure 3.**
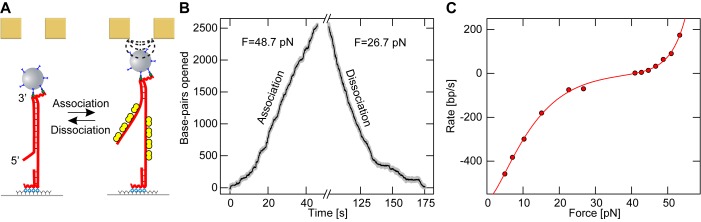
Association and dissociation of yRPA on a flap bearing DNA duplex substrate (at 20 nM yRPA in presence of 3 mM Mg^2+^). (**A**) RPA also associates reversibly onto a 6.1 kb long DNA duplex with a 38 nt long 5′-end flap subject to force. (**B**) Example time trace of RPA association and dissociation. The duplex is seen to open continuously over several thousand bp, caused by the association of RPA. In this geometry, association proceeds at a rate of 63.8 bp/s, when a magnetic force of 48.7 pN is exerted. Complete opening is avoided, to prevent detachment of the magnetic bead, by lowering the force. At a force of 26.7 pN force, the helix refolds rapidly with a rate of 70 bp/s as RPA dissociates. (**C**) Association and dissociation rates are plotted against the applied force (red circles). Again both the association and dissociation rates vary exponentially with the applied force. A double exponential (see main text) fits the data well (red line), fit Parameters are given in Table 1.

Next, we probed whether the observed RPA binding behavior is unique to the DNA hairpin geometry, where both ssDNA strands at the junction are subjected to force, or whether it could also be observed on a DNA duplex with an internal 63 nt gap and an adjacent 38 nt 5′-flap. For this substrate only one of the two DNA strands is under force at the junction, while the other strand, bearing the 5′-flap, is free from tension. In this geometry RPA molecules from the two strands at the junction are allowed to interact with each other, which may provide a different behavior. The DNA substrate utilized in this case consisted of a 6.1 kb stretch of dsDNA where the gap was 1 kb away from the 5′-end, followed by a ssDNA flap (see Materials and Methods, Figure 3A). This DNA construct underwent a rapid disruption of the base-pairing in the dsDNA when the applied force exceeds 65 pN, as indicated by a marked increase in the DNA length. This corresponds to the well established DNA overstretching transition (36,37).

In the presence of yRPA, a gradual increase in DNA length was observed at forces well below the overstretching transition. This extension corresponds to the opening of the duplex, which proceeded for several thousand bp (Figure 3B) in agreement with the association of RPA at the junction between ssDNA and dsDNA (Figure 3A and B). As observed for the DNA hairpin geometry, the process was fully reversible such that upon force reduction the DNA extension gradually decreased until full restoration of duplex DNA. Measured hairpin opening and closing rates also varied exponentially with the applied force (Figure 3C). We fit these data with Equation 1, accounting for the altered DNA stretching geometry by means of a different conversion factor *c* (Supplementary Information). The fit parameters obtained compare well with those obtained for the hairpin geometry, $\Delta z_{\mathit {{\rm on}}}=2.9\,\pm \,0.2$ bp and $\Delta z_{\mathit {{\rm off}}}=1.6\,\pm \,0.1$ bp, suggesting a geometry independent behavior of RPA binding and dissociation. Again microdomain association/dissociation appeared to govern the observed opening or closing of the duplex. The closure rate of $k_{\mathit {{\rm off}}}=564\,\pm \,11$ bp/s suggests extremely rapid exchange. An elevated force of 43 pN was required to bring both competing processes to equilibrium, which can be fully explained by considering the stretching energetics of this geometry (see Discussion).

### Magnesium dependence of RPA binding

The ionic strength and in particular the magnesium level are of vital importance for DNA-protein interactions due to both a general screening of charges and also the specific mediation of important contacts (38,39). Earlier work reported that hRPA is capable of melting dsDNA under conditions of low magnesium concentration even in the absence of force (21–23). Here, we investigated this observation in more detail for both hRPA and yRPA.

**Figure 4.**
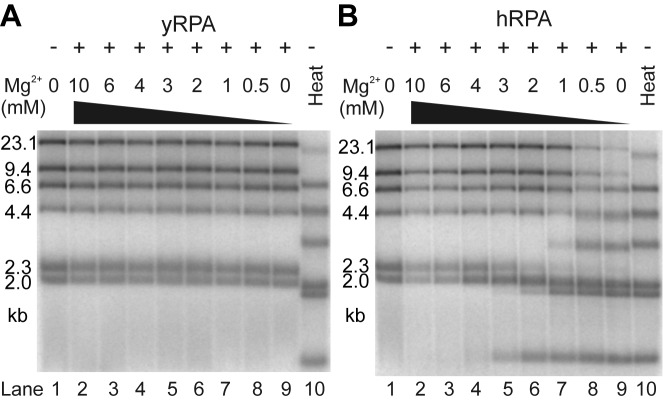
DNA melting capacity of human RPA depends on Mg^2+^ concentration. (**A**) yRPA does not melt dsDNA. Lambda/HindIII DNA (lane 1) was incubated with 2.2 μM yRPA (corresponding to 300% saturation) with various magnesium acetate concentrations (lanes 2–9, 0–10 mM as indicated) for 30 min at 30°C and subsequently analyzed on a 1% agarose gel. Throughout the range of magnesium concentrations tested, no melting occurred (*cf*. heat denatured substrate in lane 10). (**B**) Experiment as in panel a but with 2.2 μM hRPA (corresponds to 375% DNA saturation) incubated for 30 min at 37°C. In contrast to yRPA, hRPA melts dsDNA at Mg^2+^ concentrations below 3 mM (lanes 5–9).

We first probed DNA melting in bulk solution by gel electrophoresis using Lambda DNA digested with HindIII that produced dsDNA fragments of various lengths. Melting of dsDNA by hRPA occured at Mg^2+^ concentrations below 3 mM (Figure 4B). The extent of melting increased with decreasing Mg^2+^ levels (Supplementary Figure S4), increasing hRPA concentration (Supplementary Figure S3) and decreasing dsDNA fragment length (Figure 4A, Supplementary Figure S4). Surprisingly and in a remarkable contrast, no sign of dsDNA melting was observed with yRPA (Figure 4A, Supplementary Figure S3). We also tested the effect of monovalent salt on the melting activity, which was retained in 50 mM KCl and 0–1 mM Mg^2+^ (Supplementary Figure S7A) but not longer observed at 100 mM or higher concentrations of KCl (Supplementary Figure S7B).

**Figure 5.**
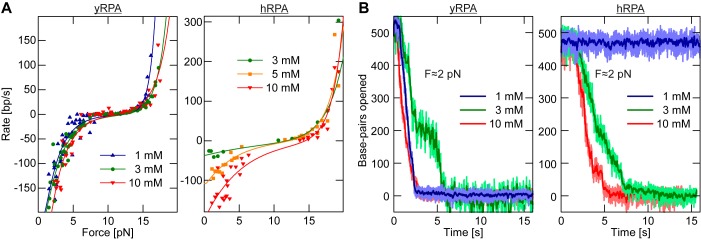
Magnesium dependence of DNA fork opening due to RPA binding. (**A**) Hairpin opening and closing rates of 20 nM yRPA (left panel) and 50 nM hRPA (right panel) as a function of force measured for different Mg^2+^ concentrations. While the behavior of yRPA is rather independent of the Mg^2+^ concentration between 1 to 10 mM, the dissociation of hRPA becomes impeded at low Mg^2+^ levels. Fit parameters are tabulated in Table 1. (**B**) Example trajectories for the dissociation of yRPA and hRPA at ∼2 pN for different magnesium concentrations. For hRPA, closure of an opened hairpin is completely impeded below 3 mM Mg^2+^.

To gain a more detailed insight into this distinct behavior of hRPA versus yRPA, we probed RPA-mediated opening and closing of dsDNA with our single-molecule assay for varying magnesium concentrations, but in the absence of monovalent salt to make the effect easier to characterize. In agreement with the gel electrophoresis experiments, the force-dependent DNA opening and closing remained constant within margins of error for yRPA in Mg^2+^ concentrations between 1 and 10 mM (Figure 5A, Table 1). In the absence of Mg^2+^, the curve is plainly shifted by 3.5 pN to lower forces, comparable to the reduction of *F*_unz_ between 1 and 0 mM Mg^2+^ (see Supplementary Figure S5). The absence of melting by yRPA is not simply due to the lower concentration of yRPA (20 nM) as compared to hRPA (50 nM). This is evidenced by the fact that hRPA melts dsDNA across a range of concentrations in bulk whereas this does not occur with yRPA (see Supplementary Figure S3).

For hRPA however, a pronounced effect of the magnesium level on the dissociation rates was observed. Hairpin closure rates at zero force dropped from 189 bp/s at 10 mM Mg^2+^ down to 109 bp/s at 5 mM Mg^2+^ and 32 bp/s at 3 mM Mg^2+^. Below 3 mM Mg^2+^ hairpin closure did not occur at all, as illustrated by exemplary time trajectories recorded at 2 pN (Figure 5B).

Even in the absence of force the DNA hairpin was already completely opened after introducing hRPA in buffer containing <3 mM Mg^2+^. Overall the observed DNA melting at low Mg^2+^ concentration in bulk and single-molecule experiments is governed by an inhibited RPA dissociation, since the hairpin opening due to RPA association was much less affected (Figure 5A, Table 1). Linear extrapolation of the hairpin closing rates for hRPA as a function of the Mg^2+^ concentration allows to estimate a value of 2.5 mM Mg^2+^ at which no more dissociation would occur. This is in good agreement with the onset of melting activity observed in bulk.

### Stepwise fork opening upon RPA binding

**Figure 6.**
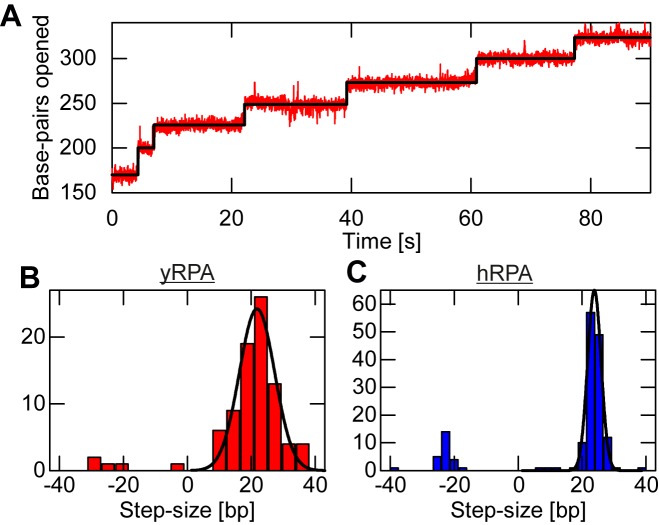
Binding site size of RPA. (**A**) Exemplary time trace yRPA association (2 nM yRPA, 10 mM Mg^2+^) on the hairpin substrate showing clearly resolved steps at a force of 12.0 pN. (**B**) Step size distribution for yRPA (from multiple traces collected under the same conditions), calculated using a step finding algorithm. The step size of 21.8 bp occurs most frequently in the data). (**C**) The step size distribution for hRPA association data (collected at (5 nM hRPA, 10 mM Mg^2+^, 12.0 pN) is similar, with the most common size being 23.8 bp.

Close examination of the progressive dsDNA opening due to RPA binding revealed that it did not occur uniformly with constant velocity, but rather in a stepwise manner (Figure 3A). To assess whether the observed stepping is introduced by the stepwise association of single RPA molecules, we carried out additional hairpin opening experiments at moderate force and low RPA concentration. These conditions favor slow RPA association such that individual steps became well resolved and discernible (Figure 6A). To evaluate the size distribution of these steps we employed a step finding algorithm (40). Step sizes for hairpin opening follow a Gaussian distribution (Figure 6B), the mean step sizes for dsDNA opening were 21.8 ± 5.5 bp for yRPA and 23.8 ± 2.1 bp for hRPA. These values are in agreement with binding site sizes of RPA on ssDNA reported in literature (26,41) in both their magnitudes (∼23 nt for yRPA and ∼26 nt for hRPA) and relative extent (with hRPA slightly larger). Thus, the observed steps appear to be single RPA binding events. Most likely, one RPA is binding to one strand and binding to the other strand then rapidly follows suit. We note that under our measurement conditions also a minor fraction of dsDNA closing steps with similar size as the opening steps were observed (Figure 6B). These backward steps stem from the dynamic competition between RPA binding and dissociation at the DNA fork.

### Sliding of hRPA along ssDNA induced by a rezipping fork

**Figure 7.**
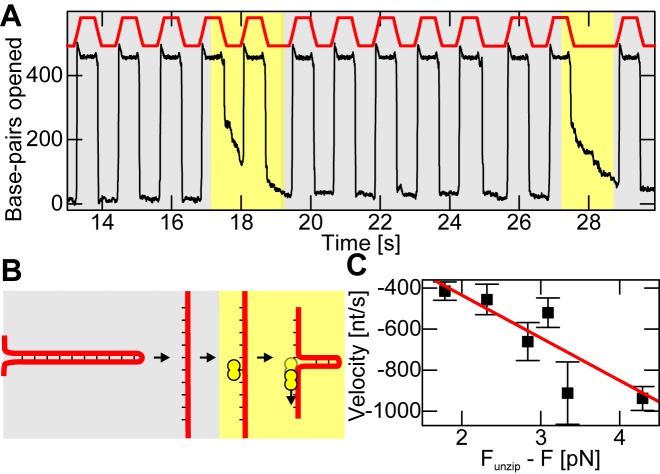
Sliding of RPA along ssDNA upon dsDNA rezipping. (**A**) Repetitive opening and closing the hairpin by alternating the applied force between 22.5 and 15.5 pN (as indicated in red) at a low hRPA concentration of 150 pM (in 1 mM Mg^2+^ buffer). For the majority of the unzipping cycles (gray background) the closing of the hairpin is unperturbed. Approximately once in 10 cycles, a continuous slower closing is however observed (pale yellow background) that is attributed to RPA sliding. (**B**) Cartoon illustrating the observed behavior according to the background colors used in A. In case of sliding events, a single hRPA heterotrimer is thought to bind to the exposed ssDNA after hairpin opening. When the force is reduced, the closing hairpin pushes the hRPA along the ssDNA. (**C**) Mean sliding velocity (black squares, error bars indicate standard errors) as a function of the pushing force applied by the hairpin (difference between characteristic unzipping force and the applied force). A linear fit describes the observed trend well (R^2^ = 0.86), with the intersection of the velocity axis at −18.23 nt/s for zero force. Furthermore, the slope of this fit can be used to calculate a friction coefficient, as the velocity is expected to vary with the force as given by *F* = ζ · $v$, for which we find a value of ζ = 0.005 ± 0.0009 pN·nt^−1^ ·s.

As shown above, at low force a rezipping hairpin rapidly displaces RPA from ssDNA. Recently, it was however reported that RPA is able to undergo diffusive motion along ssDNA (26). We therefore sought to test whether a rezipping DNA fork is able to push a single RPA heterotrimer along ssDNA in front of it. If this occurred, ssDNA secondary structure might impose pressure onto RPA filaments to keep them in a dense state. To test this hypothesis a hairpin was repetitively opened and closed by alternating between forces above and below the characteristic unzipping force (22.5 and 15.5 pN, respectively). The lower force bound allowed rapid hairpin closure in the absence of RPA in a single abrupt transition, while being sufficiently high to inhibit RPA dissociation from ssDNA. When adding small amounts of hRPA (150 pM in buffer containing 1 mM Mg^2+^) the hairpin closing transition was blocked in some instances and instead a slower rezipping with approximately constant velocity took place (Figure 7A). Such continuous closing events occurred only about once in 10 force cycles. Given that the applied lower force bound strongly disfavors RPA dissociation, we attribute these RPA induced events to the sliding of a single RPA molecule in front of the rezipping fork. RPA gets pushed along the ssDNA by the fork but slows down the rapid rezipping by the friction it experiences during sliding on ssDNA (see Figure 7B). The fact that the sliding can be interrupted by unzipping the hairpin and reinitiated by closing the hairpin again (see Figure 7A, second highlighted portion) substantiates this explanation.

To determine a friction coefficient and thus a diffusion coefficient for RPA sliding on ssDNA, we carried out RPA pushing experiments for various lower force bounds (13.5 to 16.0 pN). The effective pushing force that moves RPA along the ssDNA is the difference between the characteristic hairpin unzipping force (17.8 pN for the applied conditions) and the applied force. In agreement with friction being responsible for the slowed down dsDNA rezipping, the average hairpin closing rate was inversely proportional to the pushing force (Figure 7C). A linear fit to the data is used to obtain the friction coefficient ζ for RPA sliding along ssDNA according to *F*_push_ = *F*_unz_ − *F* = ζ · $v$. Using the Einstein relation *D* = *k*_B_*T*/ζ, the friction coefficient is converted into the diffusion coefficient *D*, for which we obtain 960 ± 350nt^2^/s.

## DISCUSSION

**Figure 8.**
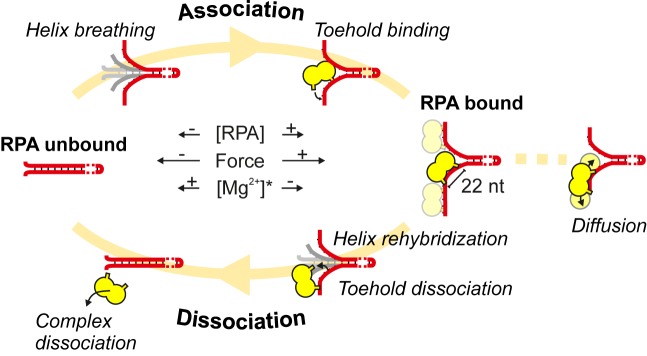
Dynamics of RPA on DNA fork. Initially, **Association** commences from a **RPA unbound** DNA fork. *Helix breathing* exposes small stretches of ssDNA to which RPA attaches via *Toehold binding*. Further binding occurs in the same manner rendering the ssDNA arms of the fork **RPA bound**. Individual bound RPA heterotrimers can slide along the ssDNA by *Diffusion*. **Dissociation** of RPA is triggered when DNA *Helix rehybridization* causes *Toehold dissociation* ultimately leading to *Complex dissociation*. Further dissociation ultimately reverts the DNA fork to the **RPA unbound** state. Both competing processes take place continuously, with the balance being controlled by the concentration of RPA, the applied force and (*in the case of hRPA) the Mg^2+^ concentration. Higher force or RPA concentration favors association, while (for hRPA) more Mg^2+^ shifts the balance toward dissociation.

RPA tightly binds ssDNA. The results presented here demonstrate that RPA binding to a DNA substrate that mimics a replication fork is, however, highly dynamic. In this case, the finely balanced competition between continuous association and dissociation of RPA at the fork determines the degree of bound RPA. The details of the dynamic model that emerges from our findings (illustrated in Figure 8) is discussed below.

### RPA supports rapid transactions at DNA forks

RPA binds to ssDNA with a dissociation constant in the low nM to high pM range (3) and dissociates with a rate as low as 0.006 s^−1^ (hRPA in 5 mM Mg^2+^) in the absence of free RPA in solution (25,42). The coating of ssDNA by RPA is therefore a rapid process, while an established RPA filament features an extremely slow turnover. In contrast, as shown in this study, RPA can be very rapidly displaced at a DNA replication fork with speeds of several hundred bp per second corresponding to a removal of up to >10 RPA heterotrimers per second. Thus, RPA acts as a versatile platform: it binds and protects ssDNA generated in a number of DNA processing steps, such as DNA repair, stalled replication and homologous recombination. During replication it supposedly also prevents rehybridization of parental DNA strands as long as a replicative helicase acts on the fork. In contrast, RPA rapidly dissociates upon rezipping of DNA at a forked substrate. Despite the stable protection of ssDNA conferred by RPA, the observed speeds (up to 300 bp/s on the fork and 500 bp/s on the flap substrate) exceed the ssDNA translocation and dsDNA unwinding rates of most helicases and translocases. Thus, RPA cannot maintain such a stretch of DNA as single-stranded and will be rapidly expelled once a helicase ceases its activity. This also implies that DNA helicases which orient themselves away from a DNA replication fork with respect to their translocation direction (28,43,44) are not required to strip off RPA but rather remove other proteins that also tightly bind ssDNA such as Rad51. An exception may be enzymes that revert stalled replication forks and that have been shown to actively anneal RPA coated ssDNA in particular in case of negative supercoiling (45). In general, helix refolding appears to be sufficient to return dsDNA to its native RPA-unbound state, once DNA processing has been completed and the processing machinery has dissociated from the DNA.

### Microdomain dynamics govern the behavior of RPA

The association and dissociation of RPA at a forked substrate was found to be strongly force dependent. Increasing forces promoted RPA association while slowing down RPA dissociation. The competition between both processes determined whether fork opening and closing was observed. The force-dependent kinetics of these processes could be described by a simple model using transition-state theory. This provided the distances from the transition states Δ$z$_on_ and $\Delta z_{\mathit {{\rm off}}}$ for association and dissociation, respectively, that were on the order of only a few bp throughout the investigated experimental conditions (Table 1). Compared to the binding-site size of RPA these values are quite small. This indicates that only a small terminal portion of RPA has to wedge in at the fork in order to allow the binding of the full RPA heterotrimer, which results in hairpin opening over the entire heterotrimer length. Similarly, for dissociation only a small terminal portion of RPA has to be lifted from the ssDNA due to helix refolding over one to two bp in order to destabilize the binding of RPA sufficiently and to shear it off. Note the transition state distances are consistently slightly lower for dissociation than for association. The mode of RPA engagement and lift off at the DNA fork is analogous to the widely used strand displacement reactions in DNA nanotechnology, where a few nucleotide long toehold provides a sufficient nucleation point for hybridization to a complementary target and displacement of an initially bound strand (35). Structural information of RPA shows that the heterotrimer comprises multiple DBDs that are flexibly linked together (46,47) and interact with the DNA in a defined orientation (48). The toehold estimates we obtain here for RPA binding compare well with the length of ssDNA that interacts with a single DBD, crystallographically determined to be 3 nt for DBDs A and B of hRPA (49). This suggests that association of a single DBD is sufficient for the observed DNA opening by RPA. Previously, such a microdomain association has already been proposed, in order to explain some of the binding properties of RPA (12,24,25). For example, the dissociation of RPA bound to ssDNA was found to be accelerated in the presence of unbound RPA in solution and it was proposed that competing RPA from solution can shear off bound molecules by initial association through a microdomain (25). Here, we directly demonstrated that RPA can melt apart and wedge into dsDNA, or once bound can be sheared off by a rezipping fork and that these processes use microdomain engagement over a length of ∼1 DBD. Given that the loss (23) or inhibition (50) of DBD-F located in the N-terminal region of the large subunit of RPA results in specific inhibition of helix-destabilization, we hypthesize that DBD-F is the likely candidate domain for toehold binding.

### DNA melting by RPA occurs without active dsDNA destabilization

When applying sufficient force, RPA is capable of opening a DNA fork. Similar to the unwinding reaction of a helicase, this could be either passive or active (51). For passive opening RPA would only interact with ssDNA and trap spontaneous helix openings (fraying of DNA duplex ends) to accomplish microdomain association. For an active unwinding RPA would also interact with the dsDNA at the fork and cause helix destabilization. The unwinding rate of a DNA hairpin under tension by a passive helicase with step size *n* has been previously derived in a simplified form as (52):

(2) \begin{equation*} v=v_{{\rm max}}\,\exp \left(\frac{\Delta G_{\rm F}^{\rm n}-\Delta G_{{\rm bp}}^{\rm n}}{k_{\rm B}T}\right), \end{equation*}

where $v$_max_ is the maximum stepping rate of the helicase, $\Delta G_{{\rm bp}}^{\rm n}$ is the average base-pairing energy over n bp in absence of force and Δ*G*_*F*_ the free energy change due to the work that is done when opening n bp in presence of force. When neglecting the entropic contribution for ssDNA stretching (52), Δ*G*_F_ = *c*_F_ · *F* · $z$_n_ and Δ*G*_bp_ = *c*_unz_ · *F*_unz_ · $z$_n_. The latter equation uses the fact that at the hairpin unzipping force the applied work exactly compensates the base-pairing energy. Inserting these two equations into Equation 2, provides an expression that is mathematically similar to the rate of force-dependent DNA opening due to RPA association (left term in Eqn. 1). For a passive helicase, the velocity approaches $v$_max_ only close to the hairpin unzipping force at which the applied tension keeps the fraying bp at the fork practically fully open, such that the forward stepping is no longer hindered by duplex formation. For an active helicase $v$_max_ is already reached at considerably lower forces (52). Given the similarities between Equations 1 and 2, we would thus expect a similar distinction to be valid also for active versus passive DNA opening by RPA. As shown above the RPA induced DNA opening velocity is monotonically increasing even when approaching *F*_unz_. Furthermore, the velocity for hRPA amounts to only 2–6 bp s^-1^ nM^-1^ at *F*_unz_ which is comparable to previously determined association rates of RPA with ssDNA of 2 s^-1^ nM^-1^ (53). Thus, the characteristics of the force-dependent DNA opening by RPA support a passive model rather than an active helix destabilization in agreement with previous reports (23). A passive model for DNA duplex opening by RPA is also supported by the observed salt dependence of this reaction for yRPA. In presence of 1 to 10 mM Mg^2+^ no considerable changes to the kinetics were found (Figure 5A). This correlates with the determined unzipping forces of the DNA hairpin that varied only slightly between 1 and 10 mM Mg^2+^ (Table 1). However, when carrying out experiments in absence of Mg^2+^ the DNA opening kinetics shifted by ∼3 pN toward lower forces, though the overall shape of the curve remained similar (Supplementary Figure S6). Again the magnitude of the shift correlates with the measured unzipping force of the hairpin, which was found to be 3.5 pN lower at these ionic conditions. A reduced base-pairing energy thus effects mechanical unzipping and RPA mediated dsDNA opening kinetics to the same measure, lending further support for the passive model.

### Fine-tuned RPA binding energetics

The above discussion established a passive mechanism for dsDNA opening by RPA. Since this process already occurs at forces below *F*_unz_, it follows that it is driven exclusively by the free energy gain of the RPA–ssDNA association to both strands at the fork. At equilibrium, when the rates of RPA association and dissociation balance out (yielding a net rate of zero), the base-pairing energy (over a single step of DNA opening of ∼23 bp) equals the work done when opening the hairpin under the external force plus the free energy change associated with RPA binding:

\begin{equation*} \Delta G_{{\rm bp}}^{23\,{\rm bp}} = \Delta G_{F_{{\rm equi}}}^{23\,{\rm bp}} + 2 \cdot \Delta G_{{\rm bind}}\nonumber \end{equation*}

Using the above expressions for Δ*G*_bp_ and Δ*G*_F_ one obtains:

\begin{equation*} \Delta G_{{\rm bind}} = (c_{{\rm unz}}F_{{\rm unz}}-c_{{\rm equi}}F_{{\rm equi}}) (23{\rm \,bp})/2\nonumber \end{equation*}

From the values in Table 1, one arrives at an average value of Δ*G*_bind_ ∼40 ± 9 kJ/mol for both yRPA and hRPA across all Mg^2+^ conditions. Using the equilibrium constant of 10^10^ M^−1^ (3), one arrives at a value of ∼57 kJ/mol which compares well to the value we obtain from our measurements. In a simplified view RPA binding contributes with about one-third of the base-pairing energy to the duplex opening, since the equilibrium force is about one third lower than the unzipping force. Remarkably this is similar for yRPA and hRPA across the range of ionic conditions investigated. In that respect RPA binding appears to be fine-tuned. The binding free energy is strong enough for stable binding but sufficiently weak to avoid DNA melting. This tunable balance is retained in 50 mM KCl between 0–1 mM Mg^2+^ but not observed at higher KCl concentrations (Supplementary Figure S7). We think that melting is meaningful within the intracellular context but probably happens on a much smaller scale. Moreover, crowding effects can shift the salt requirements for RPA-induced melting.

The obtained energetic contribution of RPA binding to DNA opening appears to be geometry independent, since also for the experiments on the DNA duplex substrate (Figure 3) the equilibrium force of 43 pN is one-third lower than the overstretching force during which DNA melts. Most likely the same contribution would be obtained when evaluating RPA induced melting of supercoiled DNA (24). However, in this case interpretation of the data may be complicated by the fact that trapping of transient denaturation bubbles may occur on multiple sites all over the DNA, as suggested by dsDNA overstretching experiments (54,55). Overall the observed force/twist-dependent DNA melting by RPA may be an important regulator in particular circumstances where DNA experiences high tension and torque. This is the case for instance during the formation of anaphase bridges in the S-phase of replication, where the affinity of the Plk1-interacting checkpoint helicase (PICH) for dsDNA greatly increases under tension causing a stabilization of the dsDNA (56).

### Regulation of RPA binding by Mg^2+^ ions

The magnesium concentration dependence of the dissociation rate we observed is specific to hRPA, yRPA did not exhibit this behavior. The ionic strength in general and magnesium in particular are of vital importance for DNA–protein interactions. While the DNA duplex is stabilized by charge screening, hindering the association of RPA, the mutual approach of negatively charged RPA and the DNA is assisted. This may account for minute variations in the apparent association rates. However, the much more significant influence on the hRPA dissociation rates seems to involve a highly specific effect of magnesium on the binding of hRPA to DNA. It is conceivable that yRPA may feature an evolutionarily conserved salt independence, in order to allow for variable magnesium concentrations in the cell. Yeast cells are expected to have greater variability of their intracellular ion composition due to the diversity of environments in which they grow. Indeed intracellular magnesium concentrations strongly depend on the magnesium concentration outside of the cell (57). Alternatively, hRPA binding could be regulated in a cell cycle dependent manner by controlling the intra-nuclear magnesium levels. Moderate magnesium concentrations are essential for the activity of a whole range of DNA repair proteins (58) and the distribution of intracellular magnesium is both variable (57,59,60) and tightly regulated throughout the cell cycle progression (58).

### Pushing of RPA along ssDNA

Despite the strong binding reported for each of the DBD subdomains, with K_d_ values in the μM range (61), diffusion of hRPA on ssDNA as been recently reported (26). We further substantiated this observation by testing whether single hRPA heterotrimers can slide along ssDNA when pushed by a closing hairpin. Sliding was readily observed and the obtained friction coefficient allowed to calculate a diffusion coefficient of *D* = 960 ± 350 nt^2^/s on ssDNA. This matches the previously reported value of *D* = 2800 ± 200 nt^2^/s (26), considering the high salt concentration of 0.5 M NaCl used in that study. Diffusion of RPA along ssDNA allows bound RPA molecules to rearrange themselves on the ssDNA, e.g. to ensure a dense coverage but also in order to free up access to other ssDNA binding proteins. It may, for example, facilitate the exchange between bound and free RPA molecules (25) and allow the recruitment of other DNA repair proteins. Rad52 for instance binds RPA–ssDNA and stimulates the extension of Rad51 filaments on the ssDNA (62–64) which is an important precursor to later stages of homologous recombination. Additionally, RPA bound to ssDNA directly interacts with ATRIP, which contributes to the activation of the ATR checkpoint kinase as a response to ssDNA arising from processing of DNA double-strand breaks and replication interference (13). *In vitro* reconstitution experiments revealed that the ATR activation is strongly dependent on the length of ssDNA, suggesting a possible cooperative mechanism of RPA–ssDNA in ATRIP mediated ATR activation (14). Therefore, the sliding of RPA along ssDNA might allow the formation of RPA nucleoprotein filaments that is optimally capable of interaction with ATRIP or proteins that might be recruited through a similar mechanism. It was recently also shown that ATR activation further depends on the binding of MutSβ to DNA hairpin loops that persist in RPA-covered ssDNA (65). In order for the formation and persistence of these hairpin regions the ability of rezipping DNA hairpins to cause RPA to slide may also be a functional requirement. Additionally, the sliding capability of RPA on ssDNA suggests that molecular motors such as helicases may also be able to push along RPA bound to the ssDNA along which they track.

## CONCLUSION

Our results contribute to the emerging view that RPA filaments are highly ‘vivid’ cellular structures. The binding affinity of RPA to ssDNA appears to be carefully adjusted, such that ssDNA intermediates arising during DNA processing are stably protected, yet dsDNA is left unchanged. When necessary, RPA can nevertheless be readily dislodged or pushed along permitting access to other enzymes. Once these molecular machines complete their task, rapid removal of RPA at a fork is facilitated by the rehybridization of the DNA helix, recovering the fully processed dsDNA.

## Supplementary Material

SUPPLEMENTARY DATA
