# Supplementary material for: Force regulated dynamics of RPA on a DNA fork
Source: Nucleic Acids Res. 2016 Mar 25;44(12):5837–48. doi: 10.1093/nar/gkw187 (PMC4937307; doi:10.1093/nar/gkw187)
Supplement: SUPPLEMENTARY DATA [file supp_44_12_5837__index.html]

Force regulated dynamics of RPA on a DNA fork — Force regulated dynamics of RPA on a DNA fork — SUPPLEMENTARY DATA 

# Force regulated dynamics of RPA on a DNA fork

## SUPPLEMENTARY DATA

- SUPPLEMENTARY DATA
