# Supplementary material for: Force regulated dynamics of RPA on a DNA fork
Source: Nucleic Acids Res. 2016 Mar 25;44(12):5837–48. doi: 10.1093/nar/gkw187 (PMC4937307; doi:10.1093/nar/gkw187)
Supplement: SUPPLEMENTARY DATA [file supp_gkw187_nar-00048-m-2016-File006.pdf]

Supporting Information

Force regulated dynamics of RPA on a DNA fork

Felix E. Kemmerich[†,1,2], Peter Daldrop[†,2], Cosimo Pinto[3], Maryna Levikova[3], Petr Cejka[3] and Ralf Seidel[1,2,*]

† - Equal contribution

[1] Molecular Biophysics group, Institute of Experimental Physics I, Universität Leipzig, Linnéstr. 5, 04103 Leipzig, Germany

[2] Institute for Molecular Cell Biology, University of Münster, Schlossplatz 5, 48149 Münster, Germany

[3] Institute of Molecular Cancer Research, University of Zürich, Winterthurerstr. 190, CH-8057 Zürich, Switzerland.

* To whom correspondence should be addressed:
Tel: +49 341 97 32501; Fax: +49 341 97 32599; Email: ralf.seidel@physik.uni-leipzig.de

Unzipping curve of the DNA hairpin substrate

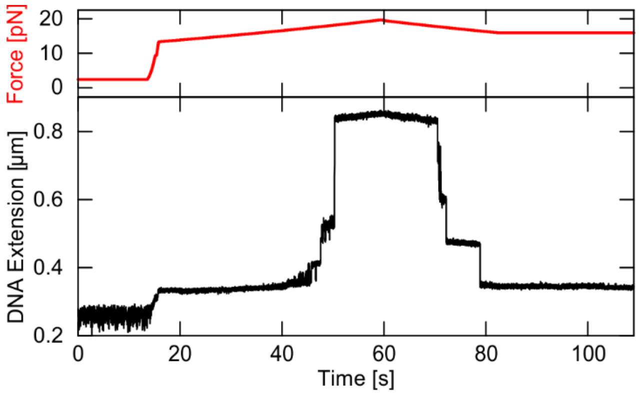

Figure S1: The DNA hairpin substrate can be mechanically unzipped

When the force applied to the DNA hairpin substrate is monotonically increased by lowering the magnets, the DNA extends in a series of sharp transitions amounting to 475 nm over the course of several seconds. The force at the final transition emanating from the intermediate state in which the hairpin is almost half open is defined is as the unzipping force, which is 18.2 pN in this case.

Conversion from DNA extension to 'basepairs-opened'

To convert DNA extensions at a given force into the corresponding number of opened base-pairs, a conversion factor was calculated for yRPA and hRPA on each construct. Force extension curves were measured for bare and RPA covered constructs. In the latter case the rehybridization was prevented conveniently by using buffer containing < 3 mM Mg^{2+} (see Main Text) for hRPA, or by annealing a short oligonucleotide. The conversion factor was then calculated by taking the difference of Freely-Jointed-Chain (FJC) fits to the force extension curves and normalization to one base-pair.

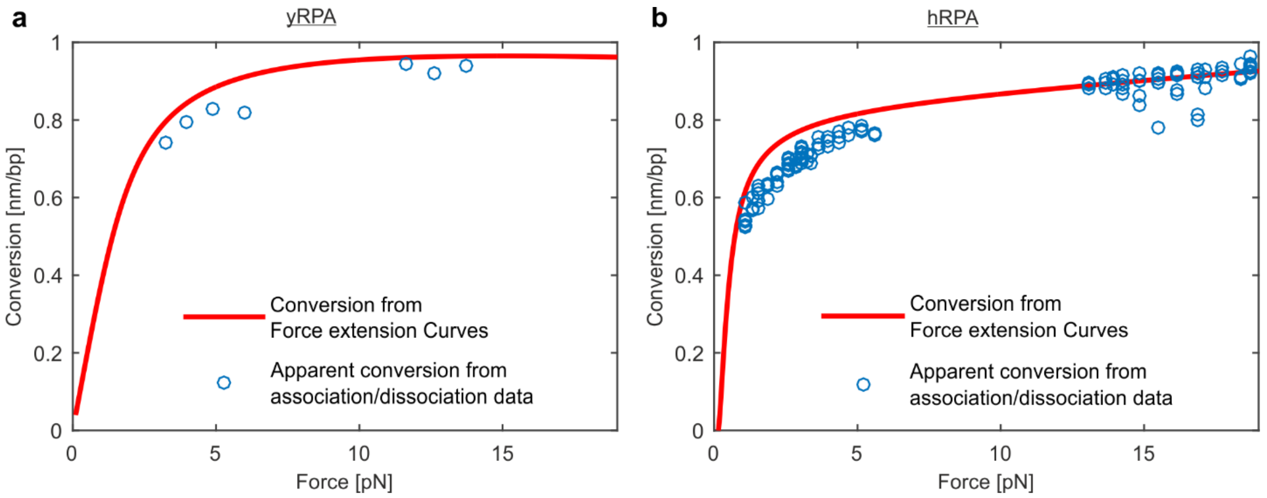

Figure S2: nm-to-bp conversion for the DNA hairpin obtained from force extension curves The conversion estimates obtained from fitting force extension curves with FJC model for **(a)** yRPA and **(b)** hRPA are shown (red lines) together with individual apparent conversions observed in the association/dissociation data. The latter were obtained from the length difference between bare dsDNA and fully RPA coated ssDNA (or vice versa) for a given force.

dsDNA Melting by hRPA

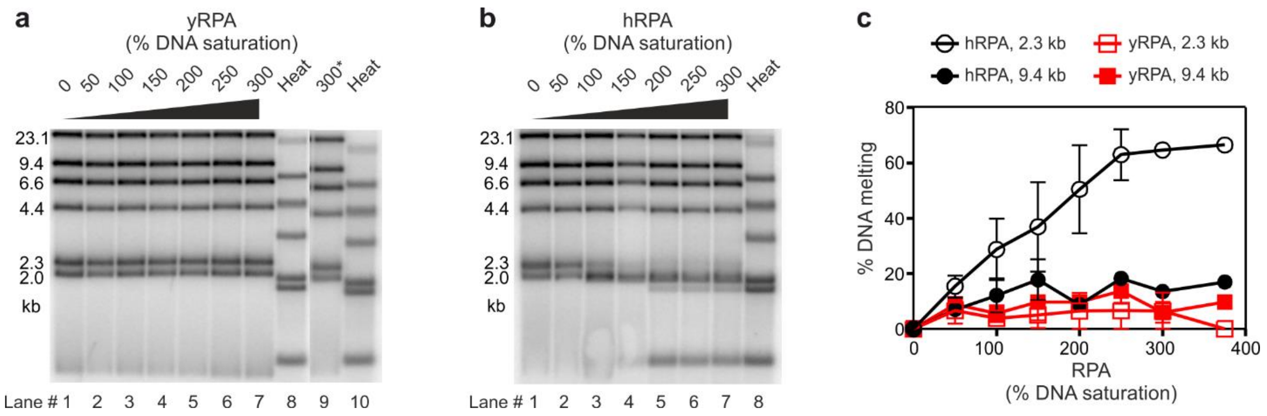

Figure S3: DNA melting capacity of human and yeast RPA

(a) yRPA does not melt dsDNA. Lambda/HindIII DNA substrate (lane 1) was incubated with various concentrations of yRPA (lanes 2-7, 50-300 % of saturation as indicated) in buffer containing 2 mM magnesium acetate for 30 minutes at 30 °C and subsequently analyzed on a 1 % agarose gel. Throughout the range of yRPA concentrations tested, no melting occurred (*cf.* heat denatured substrate in lane 8). The lack of dsDNA melting by yRPA was not due to the lower reaction temperature of experiments with yRPA (30 °C). When yeast RPA was incubated at 37 °C at 300% saturation (lane 9, *) again no DNA melting was observed. **(b)** Experiment as in panel **a,** but with various concentrations of hRPA (as indicated) incubated for 30 minutes at 37 °C. In contrast to yRPA, hRPA melts dsDNA once its concentration reaches a level sufficient for dsDNA saturation (lanes 3-7). **(c)** Quantitation for the RPA concentration dependent dsDNA melting shown in panels **b** and **c**. The 2.3 kb long dsDNA (open symbols) becomes melted to increasing extent by higher concentrations of hRPA (black), but not by yRPA (red). However, the 9.4 kb long dsDNA (filled symbols), is not melted significantly. Error bars, SEM, n=2.

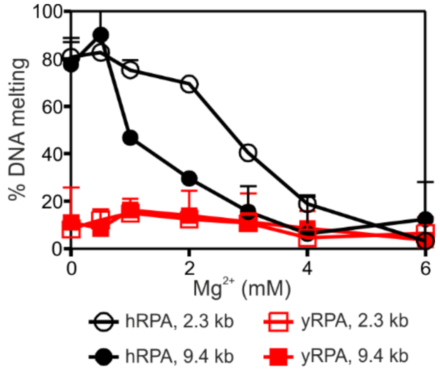

Figure S4: Quantitation of magnesium dependent dsDNA melting by RPA

Quantitation of dsDNA melting by yeast or human RPA and its dependence on the magnesium concentration (see Figure 4 of the Main Text). hRPA (black) melts both the 2.3 kb and 9.4 kb dsDNA (open and filled symbols, respectively) for magnesium concentrations below 3 mM, whereas yRPA (red) does not melt dsDNA throughout the observed range of conditions. Error bars, SEM, n=2.

Concentration dependent association of yRPA on the DNA hairpin

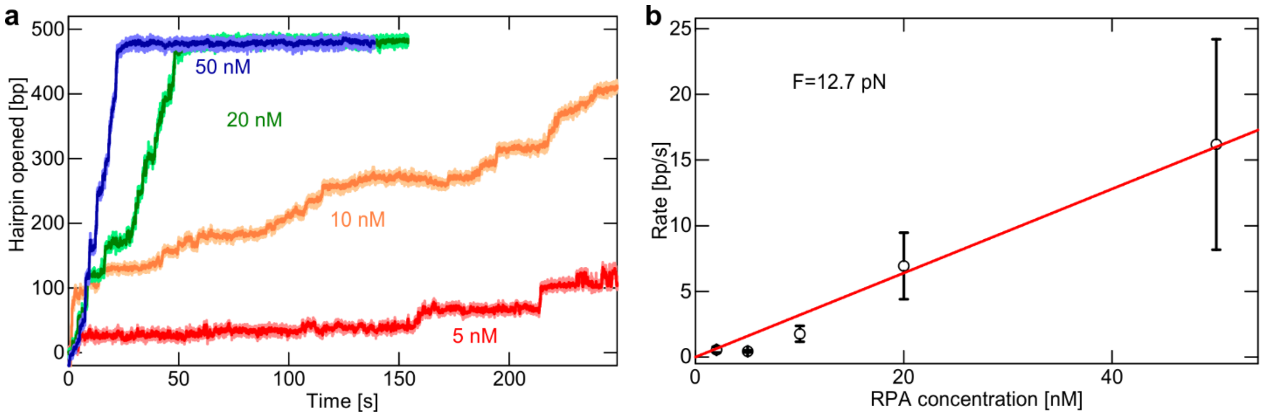

Figure S5: Concentration dependent association of yRPA on the DNA hairpin substrate
(a) Example time-traces of association at a force of 12.7 pN are shown. The rate of association is increased from 0.4 bp/s at 5 nM RPA (red curve), to 16.2 bp/s at 50 nM (blue curve). **(b)** Observed association rates (open circles) vary linearly with the concentration of RPA as indicated by the linear fit (red line). Points shown correspond to mean values of triplicate measurements with error bars representing one standard deviation.

yRPA association/dissociation kinetics in absence of Mg^{2+}

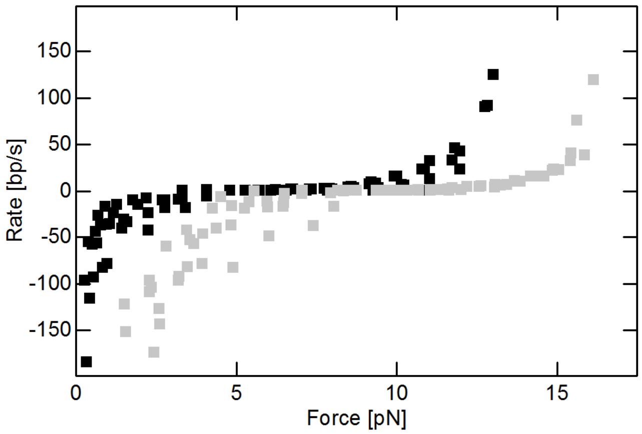

Figure S6: Salt-free association/dissociation kinetics of yRPA/
The measurement of yRPA binding kinetics were repeated in the absence of salt (black), for which we found that the entire curve is shifted towards lower forces (*cf.* the 1 mM Mg^{2+} curve in grey) while its shape is preserved. The shift is roughly consistent with the difference of 3.5 pN which we observe between the unzipping forces under these buffer conditions.

Influence of monovalent salt on dsDNA melting by hRPA

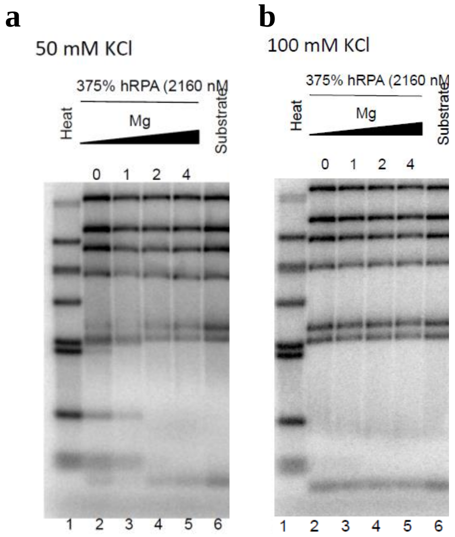

Figure S7: Effect of KCl on the DNA melting capacity of human RPA
(a) In presence of 50 mM KCl, hRPA melts dsDNA at low magnesium concentrations. Lambda/HindIII DNA substrate (lane 6) was incubated in 50 mM KCl buffer supplemented with of 0-4 mM $MgCl_2$ (lanes 2-5) for 30 minutes at 37 °C and subsequently analyzed on a 1 % agarose gel. Compared to conditions in which no monovalent salt is added, the range in which melting is observed is shifted down to 0-1 mM (lane 1 & 2, *cf.* heat denatured substrate in lane 1). **(b)** At higher concentrations of KCl the melting of hRPA is inhibited. The substrate (lane 6) was incubated in 100 mM KCl, in this case no melting was observed across the range of magnesium concentrations tested (0-4 mM $MgCl_2$, lanes 2-5, *cf.* heat denatured substrate in lane 1).
